# The use of dried tube specimens of *Plasmodium falciparum* in an external quality assessment programme to evaluate health worker performance for malaria rapid diagnostic testing in healthcare centres in Togo

**DOI:** 10.1186/s12936-020-03569-y

**Published:** 2021-01-20

**Authors:** Ameyo M. Dorkenoo, Kafui Codjo Kouassi, Adjane K. Koura, Martin L. Adams, Komivi Gbada, Gnatoulma Katawa, Kossi Yakpa, Remi Charlebois, Ekaterina Milgotina, Michele O. Merkel, Michael Aidoo

**Affiliations:** 1 Ministère de la Santé et de l’Hygiène Publique, Lomé, Togo; 2 Faculté des Sciences de la Santé, Université de Lomé, BP1515 Lomé, Togo; 3 Global Scientific Solutions for Health (GSSHealth), Baltimore, MD USA; 4 Division of Parasitic Diseases and Malaria, Centers for Disease Control and Prevention (CDC), Atlanta, GA USA

**Keywords:** Malaria, RDT, Quality control, *Plasmodium falciparum*, Proficiency testing, Togo

## Abstract

**Background:**

The use of rapid diagnostic tests (RDTs) to diagnose malaria is common in sub-Saharan African laboratories, remote primary health facilities and in the community. Currently, there is a lack of reliable methods to ascertain health worker competency to accurately use RDTs in the testing and diagnosis of malaria. Dried tube specimens (DTS) have been shown to be a consistent and useful method for quality control of malaria RDTs; however, its application in National Quality Management programmes has been limited.

**Methods:**

A *Plasmodium falciparum* strain was grown in culture and harvested to create DTS of varying parasite density (0, 100, 200, 500 and 1000 parasites/µL). Using the dried tube specimens as quality control material, a proficiency testing (PT) programme was carried out in 80 representative health centres in Togo. Health worker competency for performing malaria RDTs was assessed using five blinded DTS samples, and the DTS were tested in the same manner as a patient sample would be tested by multiple testers per health centre.

**Results:**

All the DTS with 100 parasites/µl and 50% of DTS with 200 parasites/µl were classified as non-reactive during the pre-PT quality control step. Therefore, data from these parasite densities were not analysed as part of the PT dataset. PT scores across all 80 facilities and 235 testers was 100% for 0 parasites/µl, 63% for 500 parasites/µl and 93% for 1000 parasites/µl. Overall, 59% of the 80 healthcare centres that participated in the PT programme received a score of 80% or higher on a set of 0, 500 and 1000 parasites/ µl DTS samples. Sixty percent of health workers at these centres recorded correct test results for all three samples.

**Conclusions:**

The use of DTS for a malaria PT programme was the first of its kind ever conducted in Togo. The ease of use and stability of the DTS illustrates that this type of samples can be considered for the assessment of staff competency. The implementation of quality management systems, refresher training and expanded PT at remote testing facilities are essential elements to improve the quality of malaria diagnosis.

## Background

The use of rapid diagnostic tests (RDT) contributes to achieving the World Health Organization (WHO) recommendations that all patients with suspected malaria have a confirmed diagnosis prior to anti-malarial treatment initiation. Globally, 93% of malaria cases are found in sub-Saharan Africa (SSA), with 213 million cases reported in 2018 [1]. Over 99% of malaria cases in Africa are attributed to *Plasmodium falciparum* parasite [1].

Unlike blood smear, which is considered the gold standard method and which has been used to diagnose malaria for decades, RDTs do not require specific equipment or highly skilled personnel and can be used in field settings by health workers without extensive laboratory training. Malaria RDTs, which are lateral flow immunochromatography assays, detect the presence of circulating antigens, such as HRP2 and pLDH from different *Plasmodium* species, in those who have been infected with malaria. RDTs are now the most widely used test to diagnose malaria in suspected cases, comprising 75% of all malaria tests performed [2]. The use of this type of tests allows for rapid diagnosis, which, if acted upon accordingly, can enable a reduction in time between the onset of symptoms and treatment, thus contributing to decreased transmission, morbidity, and mortality [1]. Especially when microscopy is not available or inconsistent, RDTs are an effective tool for the management of malaria.

While testing at or near the point-of-care (POC) can accelerate potentially life-saving medical decisions, procedural challenges, due in part to varied competency of health workers who conduct testing, can result in misinterpretation and misuse of RDTs, poor accuracy and misdiagnosis. Data from studies in the Democratic Republic of Congo have shown that only 18% of health workers correctly reported RDT results obtained as part of an external quality assurance (EQA) programme [3]. Unacceptably high false-positive and false-negative results from RDT use have been shown in other studies [4–6].

When RDTs are used and stored under optimal conditions (e.g. light, temperature and humidity levels), they can accurately detect malaria antigens. Recognizing that optimal conditions do not exist in many POC settings, it is important to monitor RDT usage and identify opportunities to maintain high standards of patient care.

In Togo, where malaria remains a major public health problem, children and pregnant women are disproportionately affected. The introduction of RDTs has been gradual in Togo, they were first deployed into health facilities in 2006 with expansion into community settings in 2007. As this rollout has scaled up and the associated training of personnel has been expanded, the rate of malaria cases confirmed by RDT has rapidly risen in Togo, from 0.39 million in 2010 to 0.98 million in 2018, with 2.1 million estimated cases of malaria in 2018 nationwide [1]. In 2017, rates of confirmed malaria were 233 per 1000 people with a 4% mortality rate, children under 5 years and pregnant women represented 34.6% and 3.6%, respectively, of confirmed cases [7]. External Quality Assurance (EQA), an internationally recognized system, is designed to help testers monitor how well they perform a test. Proficiency testing (PT), a form of EQA, is the process of testing quality control (QC) material containing known analyte levels (e.g. malaria HRP-2 antigen), in which testers are blinded to the composition of the QC samples [8].

Currently, there is a lack of universal, field-ready malaria QC material to test the quality and performance of RDTs, or ascertain staff competency [9]. While some malaria QC products exist, data on their use and performance is limited and are not readily available. For example, QC products using recombinant antigens do not work on all brands and types of RDTs. Therefore, the availability of high-quality, stable and affordable control material for RDTs, with broad compatibility to common RDTs, would contribute to the expansion of malaria testing [10].

Dried tube specimens (DTS), comprised of dried *Plasmodium falciparum* infected blood, have been reported to be suitable for quality control and EQA in malaria testing facilities [11, 12]. This work discusses findings from the use of malaria DTS in a PT programme in 80 healthcare centres across Togo and further highlights the need for proper quality management systems (QMS) in RDT testing.

## Methods

This was a nationwide proficiency testing survey for health workers at 80 health centres across 40 districts and six regions, selected as described below, that use RDTs to diagnose malaria cases representing a subset of all testers within Togo. These 80 health centres represent 9.6% of the total 835 health centres in Togo which use RDTs and have been accredited by the National Malaria Control Programme (NMCP). A cut off of 80 sites for this activity was necessary based on the total number of DTS vials available. The programme was performed in Togo in October 2018.

### Site selection and organization of PT activity

The PT activities were planned and implemented by the National Quality Assessment Programme Management (NQAPM) team of the Division of Laboratories of Togo Ministry of Health. Three field teams from the central level conducted the activity in the following health regions: Team 1: Lomé and Maritime health regions; Team 2: Plateaux health region and Team 3: Centrale, Kara and Savanes health regions.

**Fig. 1:**
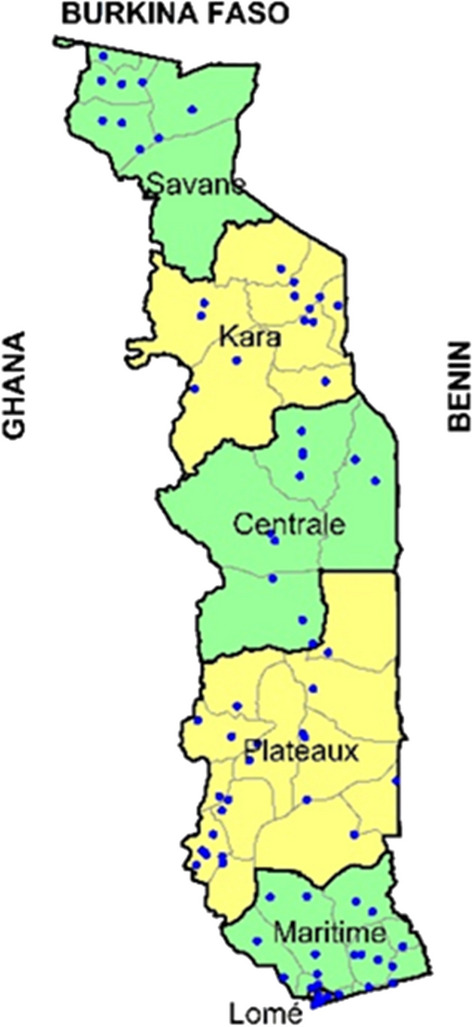
A map of Togo showing health regions and locations of the sites participating in the PT programme (blue dots)

Based on the information from the NMCP and the number of PT samples available for use, a subset, selected as described below, of the total number of health centres using RDT to diagnose malaria were selected to be included in the malaria PT activity. As sites are unevenly distributed within Togo, facilities in locations with high capacities of microscopy diagnosis (e.g. Lomé) were eliminated to give priority to locations with lower level facilities or without a dedicated laboratory. A second review of the list of sites was undertaken to identify those sites with higher throughput of patients and group them geographically to ensure representation of all six health regions of Togo while maintaining ease of access by the field teams. Finally, after applying these criteria, 80 of these sites were randomly selected as participant sites with all workers present on the day of the field team visit being evaluated. The number of facilities meeting these criteria and selected to participate in the PT programme varied across 6 regions, with Centrale Region represented by 10 facilities, Kara Region by 13 facilities, Lomé Region by 7 facilities, Maritime Region by 17 facilities, Plateaux Region by 24 facilities, and Savanes Region by 9 facilities (Fig. 1).

### Dried tube specimen preparation

DTS were prepared in the USA as previously described [11]. Briefly, a culture-adapted *P. falciparum* strain 3D7 parasite line was grown in culture and harvested when parasitaemia reached 2% (~ 100,000 parasites/µL). Parasite preparation was then diluted with washed (in incomplete medium; RPMI1640, Gibco™ Thermo Fisher, Grand Island, NY, USA) parasite negative blood at a similar haematocrit to generate preparations with 0, 100, 200, 500 or 1000 parasites/µL. Parasite negative blood was procured from a US-based blood bank and were collected from US resident donors with no reported overseas travel and confirmed by PCR to have no malaria infections. Blood was determined to be HIV and HBV negative. Blood was also confirmed in the CDC malaria laboratory to not produce a positive test on RDTs before being used to produce DTS. The diluted parasite preparations were then tested on a histidine-rich protein 2 (HRP2)-based RDT brand with performance characteristics that met World Health Organization (WHO) procurement criteria [9]. After confirmation of reactivity, the diluted parasites were distributed in 50 µL aliquots in Sarstedt® Type I microtubes (Sarstedt Inc., Newton, NC, USA) and, with the caps open, air-dried overnight in a biosafety cabinet.

To check that baseline reactivity was not affected by drying, 1 vial of DTS at each of the parasite densities was re-hydrated with a solution of PBS-Tween20 (Sigma Aldrich, St. Louis, MO, USA) and tested on the same RDTs brands as prior to drying. All DTS vials were from the same batch and stored at + 4°C before shipping via commercial air service at ambient temperatures to Togo and, similarly, were stored centrally in Lomé at + 4°C prior to field activities. As previously described, short term storage (up to 4 weeks) at ambient temperatures does not impact the reactivity of the DTS [12].

### RDT procurement and storage

A batch of SD Bioline® Malaria Ag Pf RDT (lot number 05CD094A, manufactured in Republic of Korea 09/25/2017, expiration date 09/24/2019) used in Togo was obtained from the NMCP for use in preliminary QC of DTS and in the PT. All procured kits were stored centrally until use. This allowed for the same lot number of kits to be used throughout the evaluation and provided an opportunity for baseline assessment prior to using the kits for the PT as well as eliminating the potential impact of variable storage conditions at testing sites. RDTs and DTS were provided to each health centre by the NQAPM team at the time of PT.

### Quality control (QC) of dried tube specimen at malaria reference laboratory in Togo

Before distributing DTS vials to sites, the DTS panels were evaluated by the NQAPM in collaboration with the NMCP in the malaria reference laboratory in Lomé. Six aliquots of each parasitaemia level (0, 100, 200, 500, and 1000 parasites/µl), 30 vials in total, were reconstituted in 50 µL of PBS-Tween rehydration buffer according to standard procedures provided by the malaria laboratory, CDC. Tubes were allowed to rehydrate for 1.5 h, gently shaken and 5 µL were then tested using SD Bioline® Malaria Ag Pf RDT (Standard Diagnostics, Inc., Abbott, Gyeonggi, Korea) in the same manner as patient blood samples would be tested.

### Reconstitution of the specimens

To standardize reconstitution and remove a potential source of error, all DTS were reconstituted by the NQAPM team in the field according to the procedure provided by the CDC malaria laboratory and described in previous paragraph. Each reconstituted DTS contained 50 µl of sample and thus provided sufficient sample to evaluate multiple health workers at a given site.

### Distribution of samples and site visits

To evaluate the performance of the health workers using RDTs, PT panels comprised of five reconstituted samples with different parasite densities (0, 100, 200, 500, 1000 parasites/µl) were distributed to health centres and selected laboratories. One facility received only three samples containing 0, 100, and 1000 parasites/µl due to an insufficient number of samples with parasite density of 200 and 500 parasites/µl. DTS vials were rehydrated onsite as described above. A total of 235 health workers participated in the PT activity, with three, two and one health workers testing samples in 76, three and one health facility, respectively. The number of participants at each site was a function of the number of staff on duty at the time of the supervisory visit. All personnel with a role in RDT work and present on the day of the visit participated in the PT. The NQAPM team explained the PT procedure to the health workers prior to providing reconstituted DTS. To record the observations, each evaluated health worker received a results form designed by the NQAPM. Completed forms were collected by the field teams and returned to the NQAPM for analysis.

### Statistical methods

XLSTAT Basic (version 2019, Addinsoft, France), a statistical application for Microsoft Excel®, was used for statistical analysis of data and Tableau Desktop (Version 2019.4, Tableau Software, Inc., USA) was used for data visualization. The difference between proportions was assessed using Chi-square test, followed by Marascuilo procedure to identify proportions responsible for statistically significant differences. Correlation between proportions of qualified testing personnel and regional performance was assessed using Pearson’s correlation coefficient. Paired Student’s *t*-test was used to compare the means of two paired samples. *P*-value of < 0.05 was considered significant. For the assessment of individual tester performance, PT scores were calculated as percentage of samples identified by a tester correctly (that is, negative aliquot content was tested negative and positive aliquot content was tested positive). For the assessment of health centre, district, or regional performance, correct results were aggregated as percentage of tests performed correctly at corresponding level from a total number of tests performed at corresponding level. For this analysis, a level of 80% correct PT result was selected as a value to mark acceptable proficiency. While 80% correct is used as a threshold for malaria microscopy testing and Stepwise Laboratory Quality Improvement Process Towards Accreditation (SLIPTA) 5-star certification, ideally testers would achieve 100% correct results on PT to offer appropriate patient care when conducting malaria testing.

## Results

### Quality control (QC) at the central laboratory

**Fig. 2:**
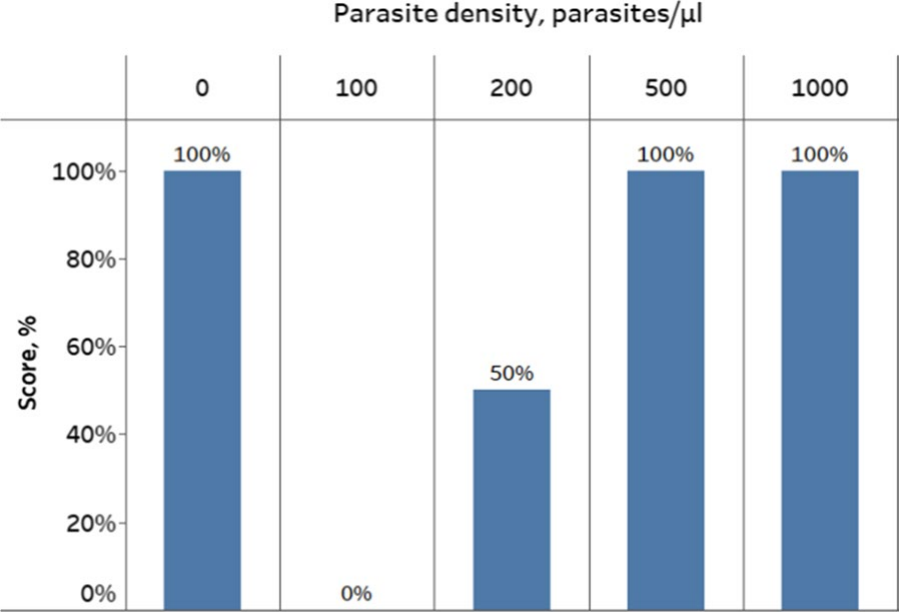
Results of QC testing (6 aliquots per parasite density) conducted at the Malaria reference laboratory prior to the initiation of proficiency testing. Data labels indicate percentage of aliquots tested correctly for each parasite density during the QC; that is, all six aliquots (100%) with parasite density 0 parasites/μl were identified as negative, none of six aliquots (0%) with parasite density 100 parasites/μl were identified as positive, only three aliquots (50%) with parasite density 200 parasites/μl were identified as positive, all six aliquots (100%) with parasite density 500 parasites/μl were identified as positive, and all six aliquots (100%) with parasite density 1000 parasites/μl were identified as positive

The results of the QC conducted at the malaria reference laboratory showed that all aliquots of DTS containing 0, 500 and 1000 parasites/µl produced expected results with the SD Bioline® Malaria Ag Pf RDT. All DTS with 100 parasites/µl and 50% of DTS with 200 parasites/µl were classified by NQAPM team as non-reactive (Fig. 2). While all parasite densities were distributed and examined during the PT programme, only 0, 500, and 1000 parasites/µl DTS samples were used for evaluation of tester’s performance.

### Overall PT programme performance

**Fig. 3:**
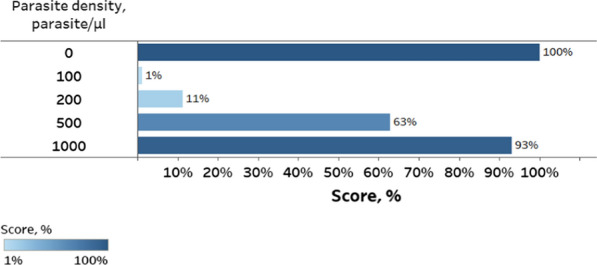
Average PT results for 235 testers across 80 participating facilities. Data labels indicate percentage of testers who correctly identified aliquot content for each parasite density during the PT; that is, all testers (100%) correctly identified aliquots with parasite density 0 parasites/µl as negative, 1% of testers correctly identified aliquots with parasite density 100 parasites/µl as positive, 11% of testers correctly identified aliquots with parasite density 200 parasites/µl as positive, 63% of testers correctly identified aliquots with parasite density 500 parasites/µl as positive, and 93% of testers correctly identified aliquots with parasite density 1000 parasites/µl as positive.

PT scores across all 80 facilities and 235 testers was 100% for 0 parasites/µl, 63% for 500 parasites/µl and 93% for 1000 parasites/µl (Fig. 3). Although health workers were not formally evaluated on their performance of testing the 100 and 200 parasites/µl DTS samples, they received those parasite densities in the PT panel and processed them. As expected and similar to the pre-study QC data, reactivity of samples with parasite density of 100 and 200 parasites/µl presented a challenge and the detection rate for samples with 200 parasites/µl was significantly lower at field sites than for the QC samples tested at malaria reference laboratory (*χ*^2^ (*df* = 1; N = 238) = 8.2; *p* = 0.004; effect size *phi* = 0.19). Forty-seven (59%) of the 80 testing facilities that participated in the PT programme received a score of 80% or higher on a set of 0, 500 and 1000 parasites/ µl DTS samples.

### Performance by healthcare worker qualification

**Fig. 4:**
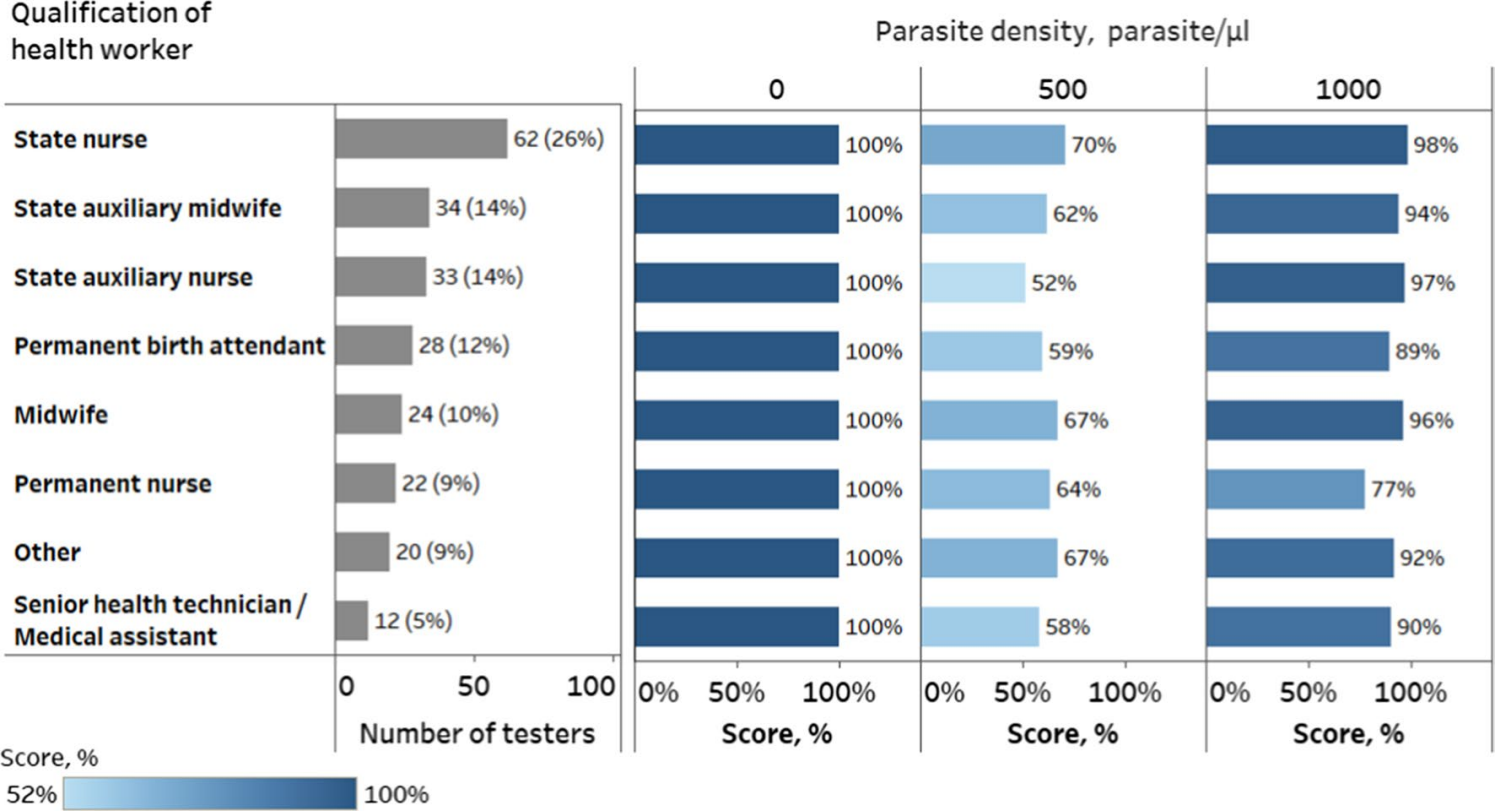
Results of PT disaggregated by health worker qualifications and DTS sample parasite density

Health workers of different levels of qualification participated in the PT programme. Ninety-one percent of health workers were classified into groups, with the majority of being state nurses, auxiliary midwives and auxiliary nurses (together 54%) while senior health technicians or medical assistants represented only 5% (Fig. 4). Other qualifications included categories with only 5 or less health workers in each and were combined into a single group identified as “Other” for group performance analysis.

All qualification groups correctly identified negative DTS and performed better in the detection of malaria in samples with parasite densities of 1000 parasites/µl than those with 500 parasites/µl (t-test for two paired samples *t *= − 9.36, *DF* = 7, *p* < 0.0001, two-tailed test) (Fig. 4). There was no significant difference between these groups of health workers in overall performance (*χ*^2 ^(*df* = 7; N = 702) = 5.6; *p* = 0.590; effect size *Cramer’s V* = 0.03) nor when comparing only the results of tests on samples containing 500 parasites/µl (*χ*^2^ (*df* = 7; N = 232) = 3.9; *p* = 0.787; effect size *Cramer’s V* = 0.05) or those with 1000 parasites/µl (*χ*^2^ (*df* = 7; N = 235) = 13.5; *p* = 0.061; effect size *Cramer’s V* = 0.09).

### Performance by health region

**Fig. 5:**
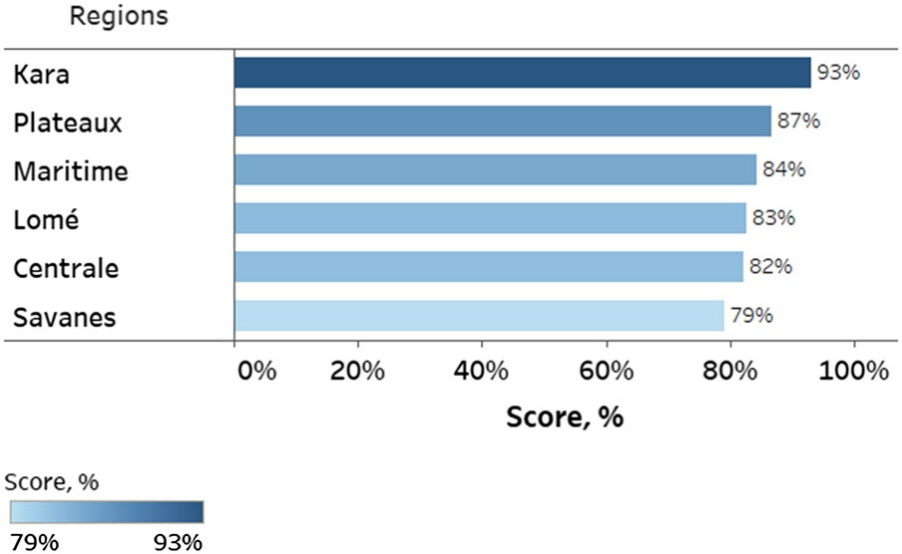
PT scores disaggregated by region. Data labels indicate regional performance score (average of all testers in that region). Performance scores were calculated using data from parasite densities of 0, 500, 1000 parasites/µl

The 80 health centres were distributed amongst multiple regions in the country (Fig. 1). The difference in PT results between health regions of Togo was not statistically significant (*χ*^2^(*df* = 5; N = 702) = 9.9; *p* = 0.078; *Cramer’s V* = 0.05) (Fig. 5). There was also no difference in performance between qualification groups within regions; and there was no correlation between the percentage of top three performing groups (state nurses, midwives and senior health technicians/medical assistants) and regional PT scores (Pearson’s correlation coefficient *r* = 0.422, *p* = 0.405) despite differences, although with small effect size, in the percentage of health workers drawn from these three groups between the regions (*χ*^2^ (*df* = 5; N = 235) = 26.4; *p* < 0.0001; *Cramer’s V* = 0.15).

**Fig. 6:**
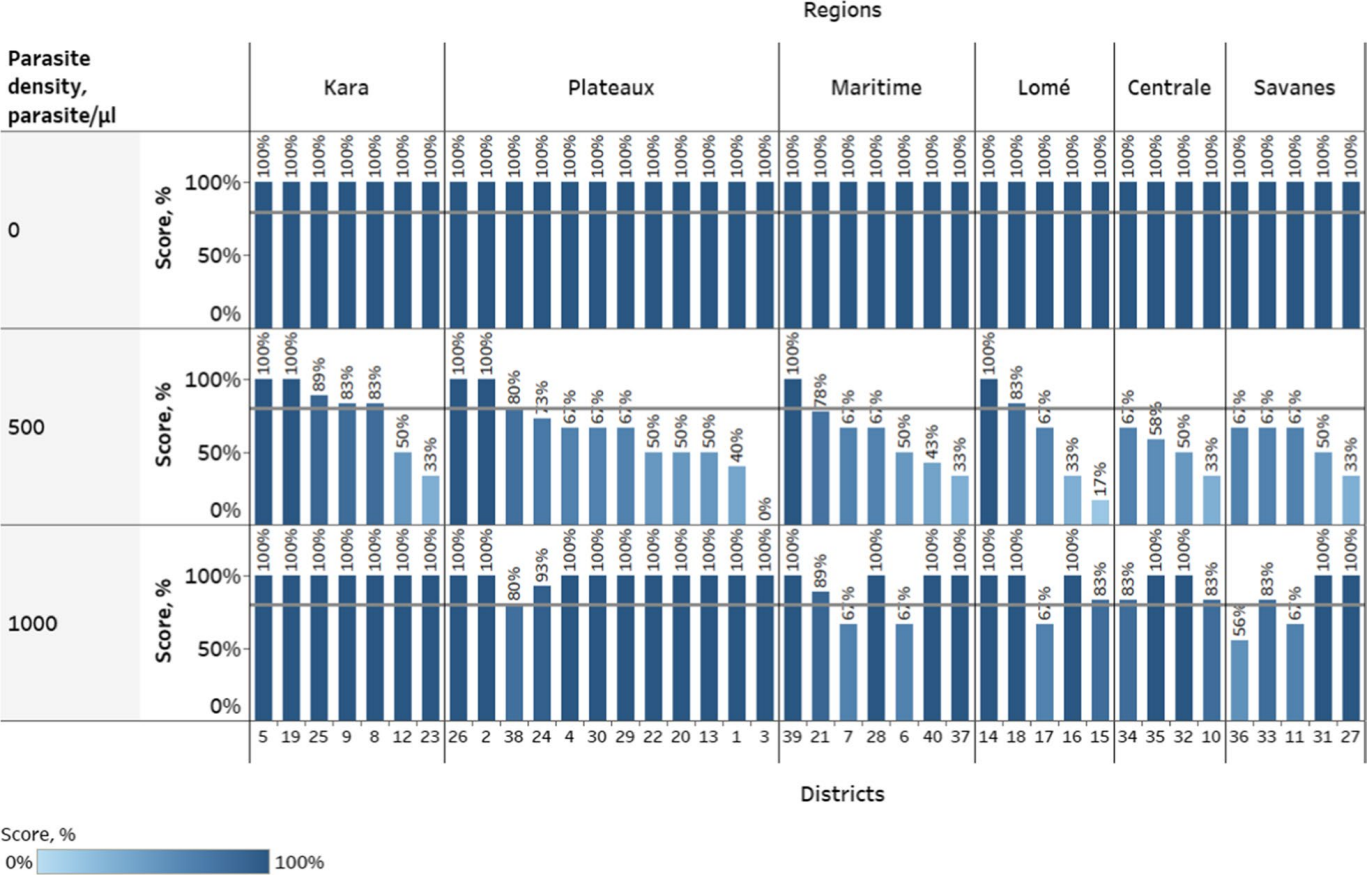
Average PT scores disaggregated by region, district, and parasite density. Data labels indicate average performance of all health workers in a given district. Horizontal grey lines indicate PT score of 80%. District numbers are arbitrary and were assigned during the study in leu of district names to anonymize sites during data analysis and to simplify the presentation of results. The difference between regions in the testing of 500 parasites/µl specimens was not statistically significant. While a difference was detected between regions in testing 1000 parasites/µl specimens, the effect size was negligible, and the data were not sufficient to identify significantly different regions.

**Fig. 7:**
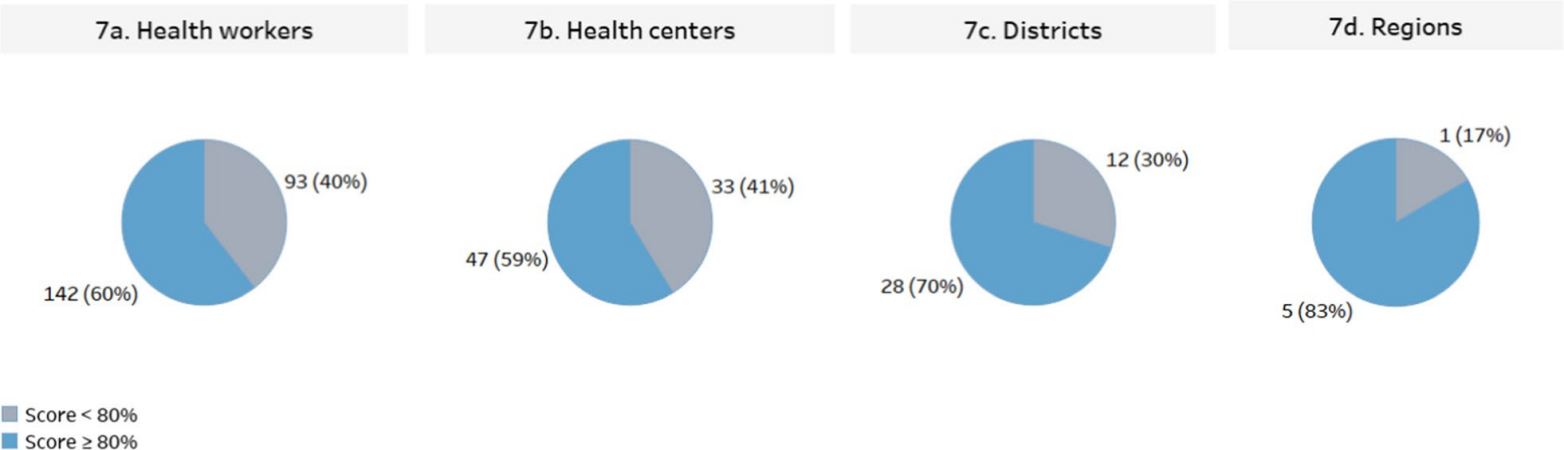
Summary of proficiency testing results across health workers, healthcare centers, districts and regions. Data labels indicate number and percentage of health workers (**a**), healthcare centers (**b**), districts (**c**) and regions (**d**)

When disaggregated by parasite density, the PT scores for 0 parasites/µl DTS samples were 100% correct across all regions. The difference between regions in the testing of 500 parasites/µl specimens was not statistically significant (*χ*^2^ (*df* = 5; N = 232) = 6.3; *p =* 0.278; *Cramer’s V* = 0.07). While a difference was detected between regions in testing 1000 parasites/µl specimens, the effect size was negligible and the data were not sufficient to identify significantly different regions (*χ*^2^(*df* = 5; N = 235) = 15.0; *p =* 0.010; *Cramer’s V* = 0.11) (Fig. 6). The six health regions are further divided into 40 health districts (Fig. 6) represented in this malaria PT by differing numbers of healthcare centres and health workers. Thirty-four (85%) and 12 (30%) of health districts had individual healthcare centres that scored below 80% for 500 and 1000 parasites/µl, respectively. Overall, 47 (59%) of healthcare centres, 28 (70%) of districts and 5 (83%) of regions performed above the threshold of 80%, and 142 (60%) of health workers correctly identified all three samples (Fig. 7).

## Discussion

While health workers who conduct diagnostic testing in the laboratory or at the point-of-care strive to operate under a well-functioning QMS, such systems are not always in place. The QMS, an ISO15189:2012 requirement, is comprised of procedures and activities that contribute to a testing system, with the goal of achieving accurate and reliable results for appropriate patient management. The use of QC material for malaria rapid testing, as a part of QMS, can be used to ascertain competency for testing. The programme described in this paper utilized DTS to assess health worker competency, through a PT programme, for performing malaria rapid diagnostic tests.

The use of DTS for PT of malaria rapid testing in this setting allowed for the successful evaluation of testers with varying levels of education at peripheral health facilities where robust QMS may not be in place. Programme administrators found that the DTS samples were easy to disseminate and transport as cold chain was not required thus minimizing costs. Malaria DTS have been found to be stable for up to 24 weeks under ambient temperature and field conditions, easing logistical challenges of handling and testing [12]. This study did not examine the impact of DTS rehydration by health workers and any errors introduced by requiring results to be returned i.e. testing without a supervisor present. While the potential impact of these activities could be relevant for large-scale programme roll-out, precedent from DTS use in HIV PT has shown that these steps can be easily incorporated into a well-planned DTS-based PT programme for malaria [12].

While the use of appropriate QC material is key to conducting PT, not all QC material is of high-quality or meets the needs of the testing programme being evaluated. During the QC step of this PT programme, it was noted that the optimal panel set consisted of 0, 500 and 1000 parasites/µl, which provided expected results on RDTs. The 100 and 200 parasite/µl DTS were not reliably detected by the RDTs during the QC step. As most RDTs on the market have a limit of detection (LOD) at or near 200 parasite/µl, RDT sensitivity is reduced at low parasite densities and cases of low sensitivity of RDTs in samples of parasite densities less than 200 parasites/µl have been reported [9, 13].

The parasite densities of 0, 500 and 1000 parasites/µl are consistent with those used by other groups for PT using malaria DTS [12]. Because most malaria tests will be reactive at 500 and 1000 parasites/µl density, any unexpected results (non-reactive) can reasonably be attributed to health worker performance rather than the test. Even though the conditions of RDT storage were not evaluated, it has been shown that improper storage of RDTs prior to use can also impact performance. For the purposes of this study, kits were delivered to sites on the day of the PT visit, thus reducing the impact of potential differences in RDT storage conditions. A limitation of the study is that despite efforts to be certain DTS produced the correct results on the RDTs, the possibility remains, especially for parasites at 500 p/µl, that some RDTs could have still produced negative results. However, based on pre-study testing data, such instances would be rare and unlikely to affect the overall result.

With the increased use of RDTs, shifting the role and location of diagnostic testing from laboratory technicians in higher-level facilities to community health workers in peripheral or point-of-care settings has become common practice. Other studies showed that community healthcare workers can successfully follow the RDTs procedural steps to diagnose malaria using RDTs when they are provided with adequate training and job materials [14, 15]. While this shift of testing to the point of care or community settings improves access to healthcare, it is important that quality and accuracy of test results are not compromised. The results of this PT programme showed that 47 (59%) of the testing facilities that participated in the PT programme received a score of 80% or higher. When probing further, low performing sites and groups of testers who scored below 80% could be identified and targeted for corrective actions. Examination of the data when arranged by regions shows 83% of the six health regions scored 80% or higher on PT, while at an individual level about 60% of testers scored 80% or more. Sites participated in the current study represent only a small subset of the nationwide testing points and these data, therefore, illustrate the need to expand PT programmes to have the greatest possible level of participation in order to truly understand the situation within a health system.

Incorrect results and incorrect interpretation of results have negative clinical implications. While no false positive results were reported by study participants, 34 (43%) of 500 parasites/µl and 13 (16%) of 1000 parasites/µl samples were incorrectly deemed negative by at least one tester at a site. At a site level, 13 (16%) of 500 parasites/µl but none of 1000 parasites/µl samples were designated as negative by all testers at a site. This creates a case for testing of samples by multiple staff members or for results to be confirmed via microscopy where personnel and reagent resources allow.

Variation in results was also seen amongst testers of different qualifications (although non-significant) and amongst different parasite density PT samples: as expected, 1000 parasites/µL DTS were more commonly identified as reactive than the 500 parasites/µL DTS. Although most clinical malaria cases are often associated with densities of 1000 parasites/µL or higher, clinical cases with lower densities are not uncommon [16–18]. Low parasite densities are associated with low test band intensities and identification of such bands is subject to tester competency and experience with such results but also to variations such as light at the testing bench and visual acuity of the tester [3, 19, 20]. This information can be useful to guide mentorship, training, and allocation of resources to maximize impact. With such information training and other corrective actions could be appropriately targeted. This study shows the feasibility of a DTS-based PT activity for malaria. The final implementation could be as described here with supervisors taking DTS to sites or as described for HIV and syphilis where DTS are shipped to sites and results returned to coordinating laboratory [21, 22].

Many factors can impact the performance of RDTs such as lack of adherence to the manufacturer’s instruction including misreading or misinterpretation of results by health workers. Errors in procedures can result in mismanaged care for patients with malaria. Such human errors include using the wrong amount or type of sample or buffer or incubating the test strip for an incorrect amount of time, especially shorter times can result in provision of incorrect test results [23]. In many settings worldwide, healthcare workers are tasked with using RDTs to diagnose malaria without receiving proper instructions on RDT use, thus underscoring the importance of high-quality training and PT [24]. The use of QC samples for PT offers data to guide further investigations to better understand the root causes of the observed errors.

## Conclusions

Using malaria DTS, the Division of Laboratories of Togo Ministry of Health was able to conduct a nationwide proficiency testing activity across multiple health worker categories. This PT programme validates the feasibility of the use of malaria DTS for PT in a field setting. The ease of preparation, dissemination, shipping and reconstitution of the DTS, along with reportable results from testers, demonstrates that DTS can serve as well-characterized, stable blinded samples for PT at parasite densities above the LOD of the rapid tests.

The PT data from Togo demonstrates that there is room for adherence to QMS and use of QC to assess health worker competency to improve malaria rapid testing across all testing settings. This programme showed that regardless of education level, the challenge for RDT users lies closer to lower parasitemia levels where minor errors in technique during testing can become much more impactful.

High-quality and routine (at minimum yearly) health worker assessments, training, site supervision and monitoring visits to follow adherence to the testing protocol can contribute to higher levels of health worker performance. These practices coupled with other QMS principles can standardize testing, thus improving patient outcomes [25, 26].

## Data Availability

The datasets generated and analysed are not publicly available but are available from the Laboratory Division of Togo Ministry of health, by the corresponding author on reasonable request.

## Abbreviations

CDC: Centers for Disease Control and Prevention
DTS: Dried tube specimen
DF: Degrees of freedom
EQA: External quality assurance
GSSHealth: Global Scientific Solutions for Health
HRP2: Histidine-rich protein 2
ISO: International Organization for Standardization
LOD: Limit of detection
MOH: Ministry of Health
RDT: Malaria rapid diagnostic test
N: Sample size
NMCP: National Malaria Control Programme
NQAPM: National Quality Assessment Programme Management
PBS: Phosphate buffered saline
PCR: Polymerase chain reaction
POC: Point-of-care
pLDH: Parasite lactate dehydrogenase
PT: Proficiency test
QC: Quality control
QMS: Quality management system
RDT: Rapid diagnostic test
SLIPTA: Stepwise Laboratory Improvement Process Towards Accreditation
SSA: Sub-Saharan Africa
WHO: World Health Organization
µL: Microlitre
